# Connectivity-Based Parcellation of the Thalamus Explains Specific Cognitive and Behavioural Symptoms in Patients with Bilateral Thalamic Infarct

**DOI:** 10.1371/journal.pone.0064578

**Published:** 2013-06-03

**Authors:** Laura Serra, Mara Cercignani, Giovanni A. Carlesimo, Lucia Fadda, Nadia Tini, Giovanni Giulietti, Carlo Caltagirone, Marco Bozzali

**Affiliations:** 1 Neuroimaging Laboratory, Santa Lucia Foundation, IRCCS, Rome, Italy; 2 Brighton and Sussex Medical School, Clinical Imaging Sciences Centre, Falmer, United Kingdom; 3 Department of Clinical and Behavioural Neurology, Santa Lucia Foundation, IRCCS, Rome, Italy; 4 Neurology Clinic, University of Rome “Tor Vergata”, Rome, Italy; University College London, United Kingdom

## Abstract

A novel approach based on diffusion tractography was used here to characterise the cortico-thalamic connectivity in two patients, both presenting with an isolated bilateral infarct in the thalamus, but exhibiting partially different cognitive and behavioural profiles. Both patients (G.P. and R.F.) had a pervasive deficit in episodic memory, but only one of them (R.F.) suffered also from a dysexecutive syndrome. Both patients had an MRI scan at 3T, including a T1-weighted volume. Their lesions were manually segmented. T1-volumes were normalised to standard space, and the same transformations were applied to the lesion masks. Nineteen healthy controls underwent a diffusion-tensor imaging (DTI) scan. Their DTI data were normalised to standard space and averaged. An atlas of Brodmann areas was used to parcellate the prefrontal cortex. Probabilistic tractography was used to assess the probability of connection between each voxel of the thalamus and a set of prefrontal areas. The resulting map of corticothalamic connections was superimposed onto the patients’ lesion masks, to assess whether the location of the thalamic lesions in R.F. (but not in G. P.) implied connections with prefrontal areas involved in dysexecutive syndromes. In G.P., the lesion fell within areas of the thalamus poorly connected with prefrontal areas, showing only a modest probability of connection with the anterior cingulate cortex (ACC). Conversely, R.F.’s lesion fell within thalamic areas extensively connected with the ACC bilaterally, with the right dorsolateral prefrontal cortex, and with the left supplementary motor area. Despite a similar, bilateral involvement of the thalamus, the use of connectivity-based segmentation clarified that R.F.’s lesions only were located within nuclei highly connected with the prefrontal cortical areas, thus explaining the patient’s frontal syndrome. This study confirms that DTI tractography is a useful tool to examine *in vivo* the effect of focal lesions on interconnectivity brain patterns.

## Introduction

The thalamus is a relay station of the brain that mediates communications between sensory, motor and associative brain regions [1]. Several previous studies in patients with thalamic lesions suggest that the thalamus, through widespread thalamo-cortical connections, is implicated in the processing of several higher level functions [2]. Anterograde memory dysfunctions have been reported to occur in the presence of damage of the anterior thalamic nuclei or the mammillo-thalamic tract (MTT) [2] [3] [4]. Conversely, lesions involving the medio-dorsal (MD), midline or intralaminar nuclei have been associated with the occurrence of executive dysfunctions [3] [5] [6]. Bilateral damage of the thalamus has been found to invariably cause memory deficits [4] [6]. Despite a large evidence for the role played by the thalamus in cognition, most interpretation of findings in terms of disconnection in neuronal circuits is based on anatomical knowledge from animal models, and remains largely speculative. In the literature, patients with similar patterns of thalamic damage are reported to exhibit patterns of cognitive deficits only partially overlapping. For instance, we recently described two cases of bilateral thalamic lesion, both showing severe anterograde memory impairment. In one case (G.P.), memory deficits were observed for both, verbal and visuo-spatial materials, while in the second case (R.F.), memory impairments were limited to verbal materials. Additionally, one patient (R.F), but not the other one (G.P.), showed severe dysexecutive syndrome. On the basis of a manual lesion definition and anatomical knowledge of thalamic nuclei and bundles, we identified, in one case (G.P.), a predominant damage of the MTT bilaterally, with a relative preservation of the intralaminar and medio-dorsal (MD) nuclei. In contrast, in the second case (R.F.), we identified a unilateral damage of the MTT (left side) associated to a bilateral damage of the midline (reuniens nucleus), intralaminar (parafascicular and central-median nuclei, CM-pf complex), and MD nuclei. We speculated that memory impairments were mainly due to a selective involvement of the MTT, while the dysexecutive syndrome was mainly driven by damage to the MD and intralaminar nuclei.

Over the last few years, diffusion magnetic resonance imaging (MRI) has become an increasingly popular tool for the investigation of structural brain connectivity in vivo. Diffusion tractography has shown the ability to reconstruct the major white matter tracts of the brain, and to provide indexes of microscopic tissue integrity (probabilistic tractography). More recently, Behrens and co-workers proposed a fully automated approach, based on probabilistic tractography, to segment the thalamus into several areas, thought to correspond to its main nuclei, based on the probability of connection between individual voxels of the thalamus and specific cortical areas [7]. This method termed “connectivity based segmentation” allows a parcellation of the human thalamus (as well as of other subcortical structures) which is broadly qualitatively consistent with mappings obtained in monkeys, as well as with volumetric measurements from cytoarchitectonic maps [8]). Using a similar approach [9], Eckert et al. reported that the MD nucleus is preferentially connected to the anterior cingulate cortex, the dorsolateral prefrontal cortex, and the caudate nucleus, while the CM-pf complex is more strictly connected to the hippocampus, amygdala, and basal ganglia.

Aim of the present study was to clarify, using connectivity based segmentation [7] whether the different neuropsychological profiles observed in two previously described cases of bilateral thalamic lesions (G.P. and R.F) can be explained by reconstructing the main patterns of connectivity and the association cortex in the healthy brain. We hypothesized that the frontal syndrome observed in one case (R.F.) but not in the other one, might be due to a selective involvement of thalamic nuclei projecting to the prefrontal cortex, known to be associated with executive functions. In the presence of macroscopic lesions, connectivity can be reduced by the decrease in diffusion anisotropy (which reduces the confidence in the estimated principal direction of diffusion). For this reason, connectivity-based segmentation of the thalamus may prove problematic in patients with thalamic lesions. We therefore decided to use a population of healthy subjects to obtain a template of the thalamic subnuclei connected to specific Brodmann areas (BAs), which we later used to identify the most likely pattern of connections likely to be affected by the lesions of G.P. and R.F.

## Materials and Methods

### Study Subjects

The clinical history of the two cases who entered the study (G.P. and R.F.) has been previously described in detail [4] [6]. Briefly, the two patients suffered from an acute ischemic event involving the thalamus bilaterally, in the absence of any detectable abnormality in the rest of the brain, without significant neurological signs. One patient (G.P.) (male, 38 year old, 17 years of formal education, lawyer) presented with a deep amnesic syndrome, characterised by a severe impairment in remembering day to day events and in learning new information. Remarkably, he showed a substantial preservation of other cognitive functions and behaviour. The second case (R.F.) (female, 61 years-old, 13 years of formal education, housewife) presented with a severe memory impairment for day to day events, together with a the dysexecutive syndrome. More specifically R.F. showed both cognitive (deficit in the planning, in the monitoring, in the attentional shifting) and behavioural disorders (dominated by inertia, apathy, lack of initiative, loss of insight, affective flattening, emotional detachment, lack of interest in her premorbid leisure and social activities). These cognitive and behavioural deficits affected R.F.’s daily living activities and her social relationships.

Both patients underwent a repetition of the same extensive neuropsychological assessment (see Table 1 for a full description) one year after their first referral to the Department of Clinical and Behavioural Neurology, Santa Lucia Foundation (Rome, Italy), two days before undergoing MRI acquisition. Nineteen healthy subjects (F/M = 11/8, median [range] age = 30 [22–40] years) were also recruited as part of a larger study designed to obtain high quality group averaged diffusion templates. These data have been previously used to compute an anatomical connectivity map template [10]. All subjects were screened by an expert psychologist to exclude the presence of any neuropsychological deficit. This research study was approved by the Ethics Committee of Santa Lucia Foundation, according to the principles expressed in the Declaration of Helsinki. Local Ethical Committee approval and written informed consent from all studied subjects were obtained before study initiation.

**Table 1:** Patients’ neuropsychological assessment.

		Case G.P.	Case R.F.	Cut-off
**General Intelligence**				
	**Raven’s Coloured Matrices** [35]	26.1	31.4	18.9
**Executive Functions**				
	**Modified Card Sorting Test** [36]			
	Criteria achieved	6	3	<6
	Perseverative Errors	0	15.4	0
	**Phonological Verbal Fluency** [35]	47.3	19.4	<17.3
	**Trail Making Test ** [**37**]			
	A	48 sec.	57 sec.	>94 sec.
	B	79 sec.	143 sec.	>283 sec.
	A–B	32 sec.	86 sec.	>187 sec.
**Short-term memory**				
	**Digit span** [38]	6.3	5.5	<3.7
	**Corsi span** [38]	5.9	4.1	<3.5
**Declarative verbal memory**				
	**15-Rey’s word list** [35]			
	Immediate recall	24.7	23.3	<28.5
	15 min. delayed recall	0	1.6	<4.7
**Declarative visual-spatial memory**				
	**Rey’s Figure** [39]			
	Immediate recall	1.0	6.8	<6.4
	Delayed recall	0.9	6.8	<6.3
**Visual-spatial abilities**				
	**Copy of Drawings ** [**35**]	7.7	9.8	<7.2
	**Copy of Drawings with Landmarks** [35]	65.1	66.7	<61.8
	**Rey’s Figure Copy** [39]	31.1	26.1	<23.7

G.P.’s and R.F.’s performance scores on tests for general intelligence, executive functions, visuo-perceptual abilities and memory functions. For the each tests, reported scores are adjusted for age, education and gender according to published normative data. References from which normative data and normality’s cut-off scores have been reported.

### MRI Data Acquisition

All subjects (patients and controls) underwent MRI brain scanning at 3.0T (Siemens, Medical solutions, Erlangen, Germany). In a single session, the following sequences were collected: (a) dual-echo spin echo (DE-SE) (TR = 5000 ms, TE = 20/100 ms); (b) fast-FLAIR (TR = 8170 ms, TE = 96 ms, TI = 2100 ms); (c) 3D T1-weighted turbo-flash magnetization-prepared rapid-acquisition gradient echo (MPRAGE) (TR = 7.92 ms, TE = 2.4 ms, TI = 210 ms, flip angle = 15°). For the dual-echo sequence, 52 contiguous interleaved axial slices were acquired with a 2 mm slice thickness, with a 192×256 matrix over a 256 mm×256 mm field of view, covering the whole brain. The MPRAGE sequence was acquired in a single slab, with a sagittal orientation a 224×256 matrix size over a 256×256 mm2 field of view, with an effective slice tickness of a 1 mm. Healthy participants only also had a diffusion weighted twice-refocused SE echo-planar imaging (EPI) sequence (TE/TR = 90/8500 ms, bmax = 1000 smm-2, voxel-size 2.3×2.3×2.3 mm3) with diffusion gradients applied in 81 non-collinear directions. Nine images with no diffusion weighting (b = 0 smm-2) were also acquired.

### Imaging Data Analysis

**Figure 1:**
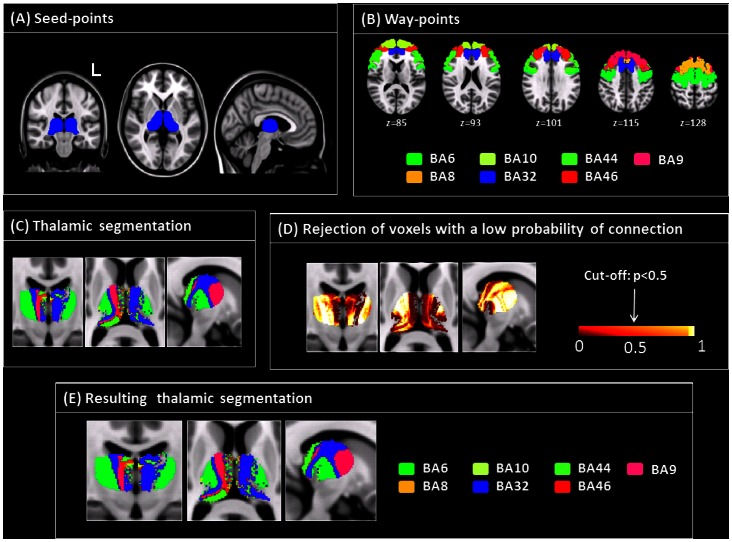
Structural connectivity maps of the thalami: processing pipeline and output. Overview of the principal processing steps used to create maps of structural connectivity between the different portions of the thalami and a selection of Brodmann areas (BAs) in the prefrontal cortex. Panel A shows the seed areas (resulting from binarizing the Oxford thalamic atlas available with FSL) used for tractography. Panel B illustrates the selection of prefrontal areas used as target points in probabilistic tractography. Panel C shows the segmentation of the thalamus in sub-regions as a function of their maximum connection probability with a given prefrontal area, before applying the minimum probability threshold. Panel D shows the map of maximum connection probability of each voxel of the thalamus with the prefrontal cortex and the threshold used to retain the highest probability connections. Panel E shows the resulting segmentation after applying the minimum probability threshold of 0.5. See text for further details.

Conventional MRI scans were reviewed by an expert neuroradiologist. Patients’ scans were compared with previous images to assess the presence of any macroscopic changes that might have occurred. Controls’ scans were carefully reviewed to exclude the presence of any macroscopic abnormality. For the purpose of the current study, the thalamic lesions were outlined by an experienced rater using the T1-weigthed images as a reference. The same guidelines followed for the previous investigation in the same patients [4] [6] were used to create masks of the lesions in both patients, using FSLview (http://www.fmrib.ox.ac.uk/fsl/). The T1-weigthed images were then warped to the T1-weighted MNI atlas (available in FSL), using the FMRIB’s Nonlinear Image Registration Tool [11], and the same transformations were applied to the lesion masks. Diffusion weighted data from healthy subjects were processed using tools from the FMRIB software library (FSL, www.fmrib.ox.ac.uk/fsl/) and CAMINO (www.camino.org.uk). After eddy current correction, the diffusion tensor (DT) was estimated voxel-wise [12], and fractional anisotropy (FA) maps were derived. First, a group-averaged diffusion tensor dataset was created in MNI space, as described in detail in [10]. Briefly, data were first corrected for eddy-current distortions and the b matrices were corrected accordingly. The diffusion tensor (DT) was then estimated voxel-wise [12] in native space, and fractional anisotropy (FA) maps were derived. The transformation (Twarp) matching every subject’s FA to the FSL FA template in MNI coordinates (1 mm3 resolution) was estimated using an affine transformation (computed by the FMRIB; linear registration tool FLIRT, [13] followed by non-linear registration using FNIRT. The same transformation (Twarp) was then applied to each component of the diffusion tensor (DT) using the PPD (Preservation of Principal Direction) algorithm [14], as implemented in CAMINO. As previously detailed [10], each component of the tensor was then averaged across subjects to yield a mean diffusion tensor dataset, which was used for probabilistic tractography. This is conceptually different from running tractography separately for each subject and then averaging the results [10]. Connectivity-based segmentation is obtained by defining the seed region (in this case, the thalamus) and the target regions (in this case, the functionally labelled pre-frontal cortices). Then probabilistic tractography is used to assign to each voxel in the seed some probability of being connected to each of the targets. The seed voxels are thus classified as connecting to the target with maximum probability, and each cluster of voxels connecting to the same target is labelled as belonging to the same substructure. For the purpose of this study, a mask of the thalamus in MNI coordinates was obtained by binarizing the Oxford Thalamic Connectivity Atlas (7, http://fsl.fmrib.ox.ac.uk/fsl/fslwiki/Atlases), thresholded at 25% probability of connection. The target areas were defined by isolating the cortices corresponding to specific BAs of the pre-frontal cortex. The masks corresponding to these BAs were obtained from the Brodmann areas template provided with MRIcron (http://www.mccauslandcenter.sc.edu/mricro/mricron/). The co-registered T1-weighted volume, also provided with MRIcron was aligned with the FSL MNI template in order to ensure a good match with the DTI data. According to the aim of the current study, the following prefrontal areas were considered: BA6, BA8, BA9, BA10, BA32, BA44, BA46 (Figure 1). These BAs are indeed implicated in cognitive and behavioural functions, which were altered in R.F but not in G.P. In particular, BA6 includes the supplementary motor area (SMA), which is implicated in actions’ internal control; BA8 has been shown to be involved in the management of uncertainty during decision making tasks; BA9 and BA46 are part of the dorsolateral prefrontal cortex (DLPFC), whose damage has been associated with impairment of executive functions, working memory, abstract thinking and intentionality, and behavioral abnormalities (i.e., apathy and inertia); BA10 (frontopolar cortex) is involved in planning, problem solving, reasoning, and episodic memory retrieval [15]. BA32 (ACC) is a relay station for processing of top-down and bottom-up stimuli [16], and is mainly involved in error detection, anticipation of tasks, attention, motivation, and modulation of emotional responses [17]; BA44 (Broca’s Area) is traditionally involved in language production, but is also implicated in working memory [18]. Next, we performed probabilistic tractography on the group-averaged DT data, using the probabilistic index of connectivity (PICo) algorithm [19] as implemented in CAMINO. PICo assigns to every voxel in the brain a probability of being connected to the seed point by considering multiple pathways emanating from the seed-point region and from each point along the reconstructed pathways. From each voxel belonging to the seed mask, we defined the probability of connection with each prefrontal BA. As we were interested in the connections between the thalamus and the prefrontal cortex only, we discarded voxels that showed a probability of connection lower than 0.5 to any of the prefrontal regions, as these voxels are more likely to be connected to other regions of the cortex. Finally, each patient’s normalised lesion mask was superimposed to the resulting map of cortico-thalamic connections to assess the most likely pattern of disconnection induced by each lesion.

## Results

**Figure 2:**
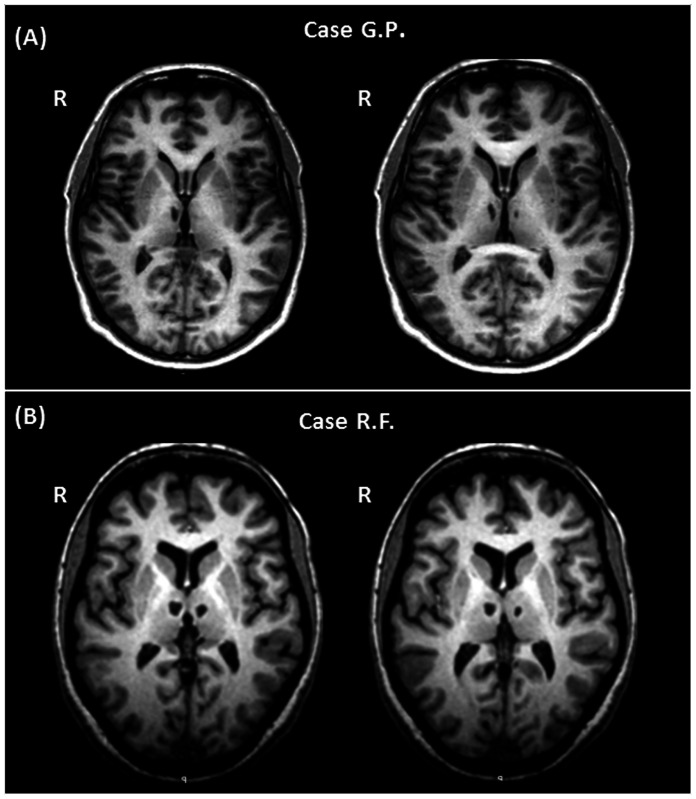
Macroscopic thalamic damage in the two studied patients. Bilateral thalamic damage detectable on T1-weighted images of the two patients. G.P. shows a more asymmetrical involvement of the thalamus, with the right lesion considerably larger than the left one (A). Conversely, R.F. presents with a more symmetrical thalamic involvement, with both lesions showing a similar location and extension (B).

**Figure 3:**
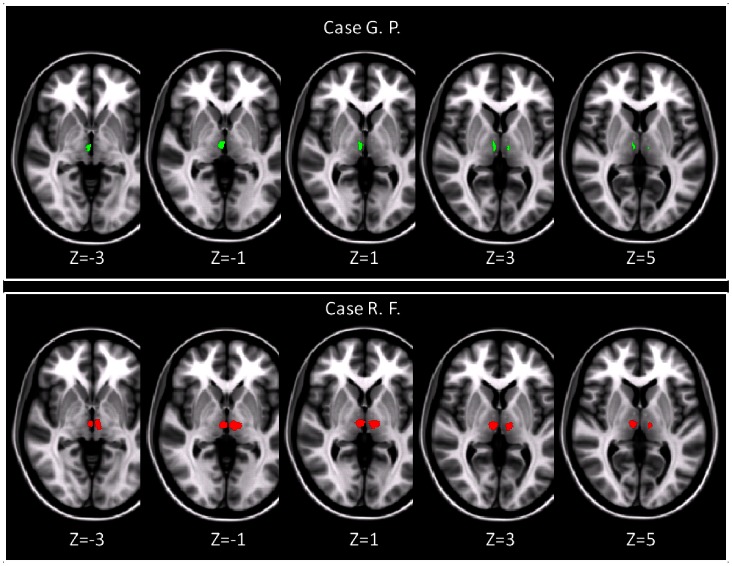
Location of the thalamic lesions after warping to MNI space. Lesion masks are shown for both, G. P. (A) and R- F. (B). The resliced lesion masks are overlaid onto the FSL T1-weighted template in standard space. The labels indicate the slice coordinate in MNI space.

**Figure 4:**
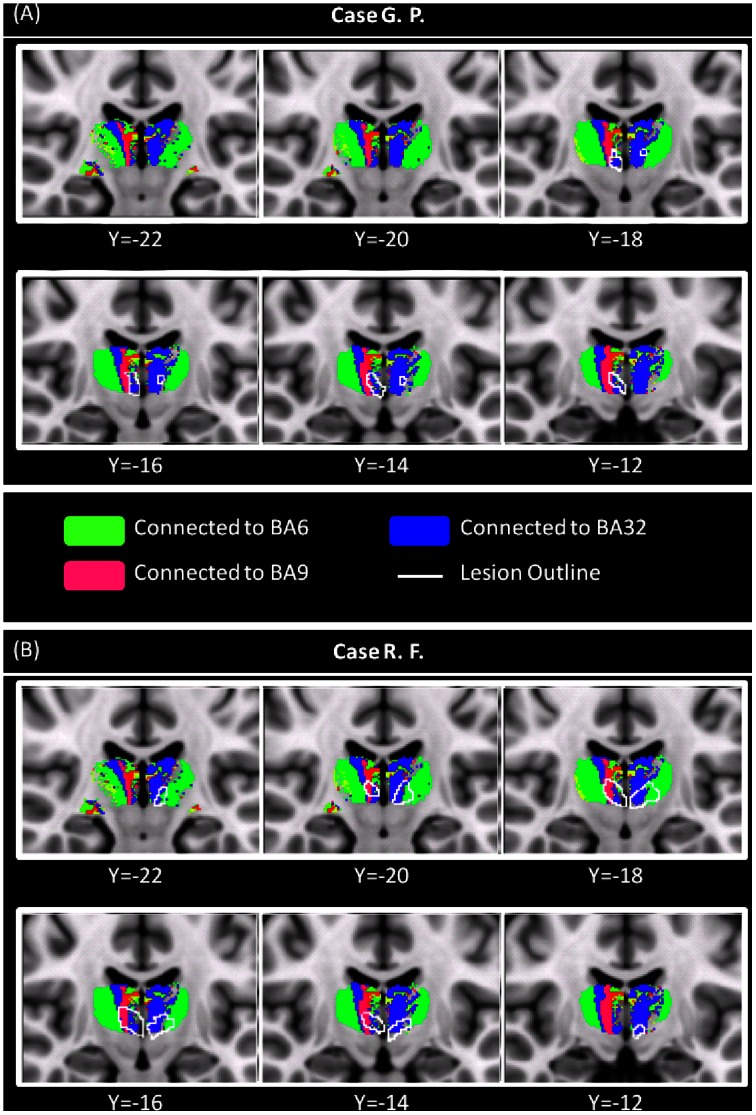
Patterns of connectivity between lesions’ voxels and prefrontal Brodmann areas. Patterns of connectivity between lesions’ voxels and the Brodmann areas of interest. Lesions outlines are shown in white, and overlaid onto the results of thalamic segmentation also shown in Fig. 1. G.P.’s lesions fell within areas of the thalamus poorly connected with the prefrontal cortex (only a few voxels belong to thalamic areas connected with the anterior cingulate cortex [BA 32]) (Panel A). R.F.’s lesions fell within thalamic areas extensively connected with several BAs of the prefrontal cortex (Panel B). In both patients, the patterns of connectivity between lesion voxels and cortical areas are consistent with the knowledge we have of thalamic nuclei projections. Abbreviations: BA = Brodmann area; CM = centro-median nucleus; IML = internal medullary lamina; MDv = medio-dorsal nucleus (ventral part); PF = parafascicular nucleus; Re = reuniens nucleus. See text for further details.

The neuropsychological assessment confirmed the cognitive profiles previously observed in G.P. and R.F. As summarized in Table 1, both patients resulted remarkably impaired at tests assessing declarative episodic memory for verbal material. G.P. also reported abnormal scores at episodic memory tests based on visuo-spatial material. On tests assessing executive functions, R.F. reported poor scores (i.e., Modified Card Sorting Test and the Phonological Verbal Fluency test), while G.P. performed normally. Visual inspection of patients’ T1-weighted images did not reveal any substantial modification with repcet to the previous scans. As shown in Figure 2 (panel A), the thalamic involvement observed in G.P. was asymmetrical, with the right lesion considerably larger than the left one. Conversely, R.F. showed a more symmetrical thalamic damage (Figure 2, panel B), with lesions in both thalami having a similar localization and extension. Additionally, R.F.’s lesions had a more posterior localization than those observed in G.P. No additional brain abnormality was detectable on patients’ T2-weighted and FLAIR scans. None of the healthy controls had any macroscopic T2- or T1-weighted abnormality. Figure 3 shows the lesion masks after warping to standard space. As shown in Figure 4 (panel A), G.P.’s lesions fell within areas of the thalamus poorly connected with the prefrontal cortex. Only a few voxels located within G.P.’s lesions belong to thalamic areas connected with the anterior cingulate cortex (BA 32). In contrast, R.F.’s lesions fell within thalamic areas extensively connected with several areas of the prefrontal cortex (Figure 4 panel B). In both patients, the patterns of connectivity between lesion voxels and cortical areas are consistent with the knowledge we have of thalamic nuclei projections. In G.P., on the left side, the few lesion voxels showing connection with BA32 are located in the area of the internal medullary lamina (IML). In R.F., lesions voxels are located in the area of MD, parafascicular and reuniens thalamic nuclei (known to project to the ACC; BA32), and in the area of CM-pf complex (known to project to the supplementary motor area; BA6), and to the dorsolateral prefrontal cortex (BA9). Table 2 summarises the percentage overlap between each lesion and these BA-connected areas defined by the connectivity-based segmentation.

**Table 2:** Percentage overlap between lesion and BA-connected areas in healthy controls.

		Lesion volume [mm^3^]	BA6-connected portionof lesion [%]	BA9-connected portionof lesion [%]	BA32-connected portionof lesion [%]
RF	LH	610	15.4	0.0	37.7
	RH	547	0.0	24.7	2.7
GP	LH	31	0.0	0.0	70.0
	RH	344	0.0	0.0	4.4

LH = left hemisphere, RH = right hemisphere; BA = Brodmann Area.

## Discussion

In this study we used connectivity-based segmentation [7] to further clarify the impact of slightly different thalamic lesions on specific cognitive functions. Both patients presented here had an acute vascular accident, resulting in an ischemic lacunar damage with a similar anatomical localization. On the basis of anatomical and functional knowledge, we previously argued that the prominent involvement of anterior thalamic nuclei and MTT might be responsible for the deficits in declarative memory (episodic and prospective memory) by disconnection mechanism with limbic cortical areas (such as anterior and posterior cingulate cortex and retrosplenial cortex) [4] [6]. We use here the term “disconnection” in the broad sense, not simply to indicate degeneration of white matter pathways, but to indicate the reduced/impaired connection between the prefrontal cortex and the rest of the brain, which might also be caused by the thalamic lesions themselves. This neuropsychological aspect was equally present in both patients. Conversely, we hypothesized that a lesion in MD and CM-pf complex might be responsible for the occurrence of a frontal syndrome [4] [6], which was observed in R.F., but not in G.P. It is known from animal and pathological studies that MD and CM-pf complex have extensive projection to the prefrontal cortex [5] [9] [20]. On this basis, we hypothesized that the damage to these nuclei might account for the additional clinical manifestations in R.F. Nevertheless, this “disconnection” based interpretation remained highly speculative and not supported by neuroimaging data. The most direct way to test for this specific hypothesis could have been the acquisition of diffusion data from our patients, and the tractographic reconstruction of fibers passing through each thalamic lesions. However, this approach suffers from at least two major technical limitations. First, the FA values within ischemic lesions are dramatically reduced, thus increasing the uncertainty around the principal direction of connections and making any result of diffusion tractography unreliable. Second, both patients were in a chronic phase of their ischemic event, and white matter fibers passing through the lesions are likely to have undergone degenerative processes. To bypass these issues, we first defined, by connectivity-based segmentation in a group of healthy subjects, the probability of structural connectivity between each voxel of the thalamus and the areas of the prefrontal cortex mainly involved in executive functions (BA6, BA8, BA9, BA10, BA32, BA44, BA46). Then, we overlaid the thalamic lesions of the two patients (G.P, and R.F.) to the connectivity atlas, and we assessed the strength of connectivity between each voxel included in the thalamic lesions and the prefrontal cortical areas. As expected, G.P.’s thalamic lesions fell within thalamic regions that are poorly connected with prefrontal cortices, thus explaining the absence of frontal deficits in this patient. According to our segmentation, G. P.’s lesions were located in areas of the thalamus with a higher of being connected to BA32 and to the IML than to other frontal areas. The IML is a “Y” shaped bundle of fibers that runs the anterior-posterior length of thalamus, and divides it in its medial and lateral part. Previous lesion studies in humans [21] [22] and animal models [23] [24] have consistently reported an involvement of the IML in learning and memory processes. Further, BA 32 has been shown to be relevant for prospective memory abilities [25] which were impaired in G.P., as previously reported [6].

On the other hand, R.F.’s lesions were mainly located in voxels highly connected with several prefrontal areas, including the anterior cingulate cortex bilaterally (BA32), the right dorsolateral prefrontal cortex (BA9) and the left supplementary motor area (BA6). These rich thalamo-cortical connections may account for the dysexecutive syndrome (characterised by poor performances at test evaluating executive functions, apathy, inertia and flattened affect) that specifically characterized R.F., but not G.P. When looking at R.F.’s lesion locations, they appear to involve the MD and CM-pf complex bilaterally. In support to our disconnection interpretation for the frontal deficits observed in R.F., a recent DTI-based tractography study in macaques and humans [20] revealed the existence of strict connections between MD and the prefrontal cortices. In more detail, the medial part of MD was found to be preferentially connected with the lateral orbito-frontal cortex; the caudo-dorsal part of MD with the medial prefrontal and cingulate cortex; the lateral part of MD with the lateral prefrontal cortex. In addition, several neuropsychological studies have suggested a strict association between prefrontal integrity and maintenance/switching of cognitive sets [26] [27], thus reinforcing the idea that MD plays a critical role in executive functions. Finally, due to its connections with the dorsolateral pre-frontal and cingulate cortex, lesions in the MD may account also for the apathy and the inertia syndrome observed in R.F. [28] [29] [30]. More specifically, our results are in accordance to Krause and co-workers [30]. These authors have recently demonstrated, in a large cohort of patients with thalamic infarcts and apathy, a disruption in the fronto-subcortical networks involving mediodorsal thalamic nuclei and both orbito-frontal and anterior cingulate cortex.

We would like to reiterate that these data do not attempt to demonstrate an involvement of white matter pathways in these two patients, but merely to support the likely pattern of connections between the thalamic lesions they presented and specific pre-frontal areas. This approach differs from the one we used in our previous work [4], which was based on a manual outline and identification of thalamic nuclei based on T1-weighted images, because the method used here is fully automated and therefore unbiased.

The results of the connectivity-based segmentation cannot be compared with previous examples, as this approach is more typically used to perform a segmentation of the whole thalamus and not just of its frontally connected portion. One interesting observation is that our results suggest a marked left to right asymmetry in healthy subjects. This finding is consistent with previous demonstrations of hemispheric asymmetry in patterns of both structural [31]–[32] and functional [33] connectivity.

The approach described in this paper has several limitations. First, the template used to estimate the segmentation of the thalamus was obtained from data obtained from young adults (between 20 and 40). While this age range includes G.P.’s age, R.F. is significantly older. It is difficult to predict how age affects the segmentation of the thalamus. A recent investigation [34] showed that the volume of the thalamus is significantly reduced with age, also suggesting a significant decrease in the volume of the thalamo-frontal projections. Further studies are required to assess the impact of these physiological changes on connectivity-based segmentation. Second, the model of diffusion used is a single tensor, which is unable to account for crossing or branching fibres. Although this might have consequences on the results of the segmentation, it is the same approached described in [7] and [8]. Third, as observed in [10], the choice of building the template from the averaged tensor data can remove intra-subject variability. Finally, the segmentation is based on probabilistic tractography, and therefore suffers from all the limitations of this technique. In particular, the probability of connection is strongly affected by the distance between the seed and the target, and thus it is possible that some cortical regions, located farther than others from the thalamus (e.g., BA10), show lower probability of connection due to this reason.

## Conclusions

In conclusion, the current investigation provides support to the disconnection mechanism (in the broad sense) as a contributor in determining cognitive and behavioural deficits in patients with focal thalamic lesions, making our previous interpretations less speculative [4] [6]. In a more general perspective, this study proposes a novel strategy to assess in single patients with focal lesions their broader effects due to disconnection mechanism, even in the case of damage of highly integrative brain structures, such as the thalamus.
